# Transmission of human hepatitis C virus from patients in secondary cells for long term culture

**DOI:** 10.1186/1743-422X-2-37

**Published:** 2005-04-19

**Authors:** Dennis Revie, Ravi S Braich, David Bayles, Nickolas Chelyapov, Rafat Khan, Cheryl Geer, Richard Reisman, Ann S Kelley, John G Prichard, S Zaki Salahuddin

**Affiliations:** 1 Department of Biology, California Lutheran University, Thousand Oaks, California, USA; 2 California Institute of Molecular Medicine, Ventura, California, USA; 3 Institute of Molecular Medicine & Technology, Huntington Hospital, Pasadena, California, USA; 4 Center for Women's Well Being, Camarillo, California, USA; 5 Community Memorial Hospital, Ventura, California, USA; 6 Ventura County Hematology-Oncology Specialists, Oxnard, California, USA; 7 Ventura County Medical Center, Ventura, California, USA; 8 University of Southern California, Los Angeles, California, USA

## Abstract

Infection by human hepatitis C virus (HCV) is the principal cause of post-transfusion hepatitis and chronic liver diseases worldwide. A reliable *in vitro *culture system for the isolation and analysis of this virus is not currently available, and, as a consequence, HCV pathogenesis is poorly understood. We report here the first robust *in vitro *system for the isolation and propagation of HCV from infected donor blood. This system involves infecting freshly prepared macrophages with HCV and then transmission of macrophage-adapted virus into freshly immortalized B-cells from human fetal cord blood. Using this system, newly isolated HCV have been replicated *in vitro *in continuous cultures for over 130 weeks. These isolates were also transmitted by cell-free methods into different cell types, including B-cells, T-cells and neuronal precursor cells. These secondarily infected cells also produced *in vitro *transmissible infectious virus. Replication of HCV-RNA was validated by RT-PCR analysis and by *in situ *hybridization. Although nucleic acid sequencing of the HCV isolate reported here indicates that the isolate is probably of type 1a, other HCV types have also been isolated using this system. Western blot analysis shows the synthesis of major HCV structural proteins. We present here, for the first time, a method for productively growing HCV *in vitro *for prolonged periods of time. This method allows studies related to understanding the replication process, viral pathogenesis, and the development of anti-HCV drugs and vaccines.

## Introduction

The global public health impact of chronic HCV infection and consequent liver disease continues to grow in numbers. It has been estimated that there are over 170 million carriers of HCV worldwide, with an increasing incidence of new infections [1]. In the United States, an estimated 1% to 5% of the 2.7 million individuals that are currently chronically infected will die due to the HCV infection [2].

Although HCV has proven to be very difficult to grow *in vitro*, HCV-RNA has been detected in cell cultures of a variety of cell types, the presence of positive-strand HCV-RNA persists for periods ranging from a few days to several months, albeit with no evidence of infectious virus [3-6]. The recent creation of HCV-RNA replicons has contributed to a better understanding of some of the molecular events, particularly gene expression [7-9]. However, studies using parts of a virus can only give limited insights about the infectious process and pathogenesis of a specific genotype. For the development of effective rational therapies and the production of protective vaccines, a reproducible *in vitro *system for the isolation and replication of HCV from patients is critical.

We report here that the isolation and long-term replication of HCV *in vitro*. Since this is the first experience with actively replicating HCV *in vitro*, some of the results shown here may not fit the current concepts using systems that do not replicate infectious virus.

## Materials and Methods

### Infection of cultured cells with sera from HCV infected patients

HCV infected patient serum (minimum of 10^4 ^genome equivalents/ml) was filtered through 0.45 μ filters (Fisher Scientific) and frozen in 1 ml aliquots at -70°C. A fresh vial of frozen serum was used for every new transmission experiment. The cells were infected using 500 μl of thawed donor serum [10,11].

### Generation of macrophages

Macrophages were generated from human cord blood mononuclear cells (CBMCs) by treating with Phorbol-12-myristate-13-acetate (PMA, 5 ng/ml in complete medium) [12]. A majority of the cells that adhered to the plastic were positive for non-specific esterase and phagocytosis, which are established markers for all macrophages. Multiple flasks (Falcon 3108 and 3109) were prepared in all cases to be used separately either for infection with HCV sera or for coculture with the infected patient's peripheral blood mononuclear cells (PBMC). The non-adherent cells contained approximately 60% CD19 and CD20 positive B-cells, with T-cells and monocytes accounting for the remainder. The cells that did not stain for macrophage-specific markers or phagocytosis were designated as non-committed lymphoid cells, and then infected either with HCV using 500 μl sera or cocultured with PBMC from the same patient.

### Infection of macrophages with HCV

**Figure 1:**
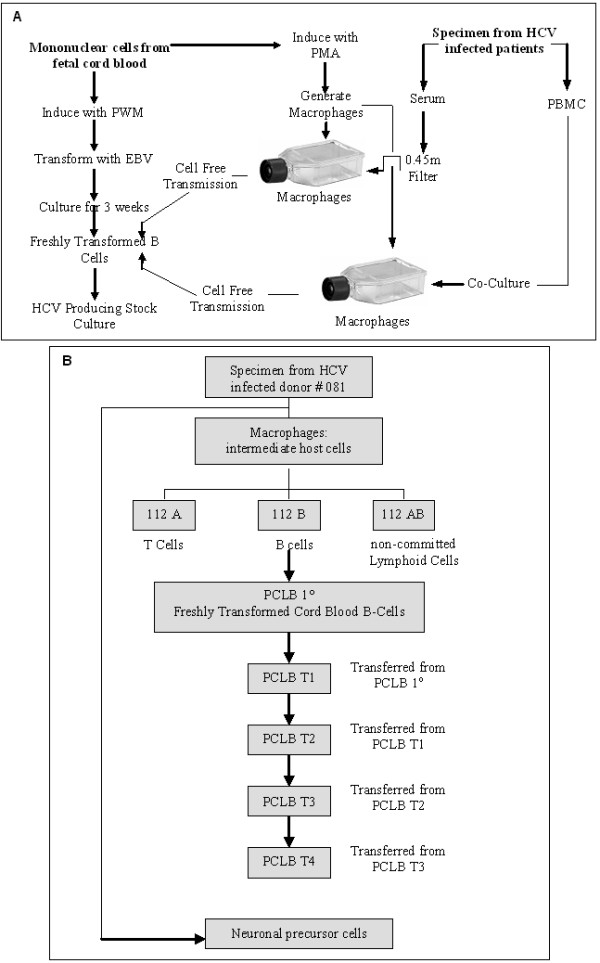
**Isolation of HCV from human patients**. (**A**) Isolation scheme for the replication of HCV *in vitro*. (**B**) History of transmission of the specimen donated from HCV infected patient #081. Fresh macrophages were infected by using cell-free serum or cocultured with HCV infected PBMC from the blood of patient #081. Human T-cells (112 A), B-cells (112 B) or the non-committed lymphoid cells (112 AB) were then either infected by cell-free transmission of HCV from cell culture supernatant from macrophages or cocultured with HCV infected macrophages. Similarly freshly transformed cord blood B-cells (PCLB 1°) were infected by cell free transmission from previously infected B-cell (112 B) culture supernatant. Uninfected transformed B-cells (PCLB T1-T4) were infected by serial, cell-free transmission from filtered PCLB 1° culture supernatant. Neuronal precursor cells were infected by cell free transmission of HCV from filtered #081 culture supernatant.

The macrophages were first treated overnight with polybrene (5 ng/ml) and then infected either with 500 μl of sera or cocultured with the PBMC from the same patient (Fig. 1A). These infected macrophages were incubated overnight at 37°C in a 5% CO_2 _atmosphere. Media were changed and the cultures were continued for another six days with change of media on day four.

### Generation of immortalized B-cells

To create immortalized B-cells, cord blood mononuclear cells (CBMC) were stimulated with pokeweed mitogen (PWM, 5 μg/ml in complete culture medium), and then infected with transforming Epstein-Barr virus (EBV). These immortalized B-cells did not produce EBV [13,14].

### Preparation of cell culture supernatants

Media taken from the cultures of infected macrophages were centrifuged at 500 × g for 10 minutes. The supernatants were then filtered through a 0.45 μ filter to remove extraneous material. The filtered supernatant is referred to as the cell culture supernatant.

### Cell free transmission of HCV

The target cells were pretreated overnight with polybrene (5 ng/ml). A 500 μl aliquot of cell culture supernatant was used for infecting each of the target cells.

### Design of positive- and negative-strand primers

In order to identify HCV-RNA, nested primers for each strand from the 5' untranslated region (UTR) were designed by CIMM using the default parameters of the DNASTAR PrimerSelect program (Table 1).

**Table 1:** Primers used to analyze HCV

**Primer**	**Strand**	**Sequence (5' to 3')**^1^
HCV 9.1	positive	gac act cca cca tag atc act c
HCV 9.2	positive	cat gat gca cgc tct acg aga c
HCV 10.1	positive	ctg tga gga act act gtc ttc acg cag
HCV 10.2	positive	cac tcg caa cca ccc tat cag
HCV 1	negative	act gtc ttc acg cag aag cgt cta gcc at
HCV 2	negative	cga gac ctc ccg ggg cac tcg caa gca ccc
HCV 3	negative	acg cag aaa gcg tct agc cat ggc gtt agt
HCV 4	negative	tcc cgg ggc act cgc aag cac cct atc agg
HutLA2	positive	ggg ccg ggc atg aga cac gct gtg ata aat gtc

^1^The primers were designed with the program PrimerSelect (DNASTAR) using conserved HCV sequences downloaded from GenBank.

### Detection of positive- and negative-strand HCV-RNA by nested RT-PCR assay

Total RNA was extracted from infected cell culture supernatants harvested 5 days after a change of media (Tri Reagent LS, Molecular Research Center Inc. Cincinnati, OH). A 269 base pair region was amplified by nested RT-PCR from the highly conserved 5'-UTR of the HCV genome.

The positive strand assay was performed using a 10 μl aliquot of the total extracted RNA was reverse transcribed using the primer HCV 9.2 with the MMLV Reverse Transcriptase (Promega Corp. Madison, WI) or with the Sensiscript Reverse Transcriptase (Qiagen Inc. Valencia, CA) according to the manufacturers' instructions. A 5 μl aliquot of the cDNA was then amplified by nested PCR using HCV 9.1 and HCV 9.2 as the outside primers, followed by amplification of 5 μl of the first PCR product using HCV 10.1 and HCV 10.2 as the inner primers.

The negative strand assay was performed by using the Oligotex Direct mRNA purification kit (Qiagen Inc.) to extract RNA from the cells. A 10 μl aliquot of the RNA was reverse transcribed using the HCV1 primer with the Thermoscript Reverse transcriptase (Invitrogen) according to manufacturer's instructions. Nested PCR amplification was then carried out on a 5 μl aliquot of the cDNA using HCV1 and HCV2 as the outer primers, followed by amplification of 5 μl of the first PCR product using the HCV3 and HCV4 as the nested primers under standard PCR conditions.

For each PCR, forty cycles of amplification were performed with the following temperature profiles: 94°C for 1 min, 55°C for 1 min, and 72°C for 1 min for the outer primer set and 94°C for 1 min, 60°C for 1 min, and 72°C for 1 min for the inner primer set.

### Detection of positive-strand HCV-RNA by Real-time RT-PCR

The total extracted RNA was solubilized in 10 μl of RNase-free water and then reverse transcribed using the primer HCV 10.2 with the MMLV Reverse Transcriptase. A 5 μl aliquot of the cDNA was then amplified by real-time PCR, using HCV 10.1 and HCV 10.2 primers on the Rotor-Gene 200 amplification system (Corbett Research, Australia) and the SYBR Green I fluorescent dye (BioWhittaker Molecular Applications, Rockland, ME), using the manufacturers' instructions. An *in vitro *transcribed RNA from the HCV 5'-UTR was utilized as the standard. Forty cycles of amplification were performed with the following temperature profile: 94°C for 1 min, 55°C for 1 min, and 72°C for 1 min.

### Detection of HCV-RNA by *in situ *hybridization

Approximately 6 × 10^4 ^cells were centrifuged (Cytospin II, Shandon, Pittsburgh, PA) onto RNase-free Poly-L-lysine coated slides (Fisher Scientific, Pittsburgh, PA), forming a uniform well spread monolayer of cells. These cells were fixed and desiccated with ethanol. Cells were then rehydrated with 1× SSC buffer and treated for protein digestion with proteinase K (Fisher) for permeation and retention. Hybridization of the probes to the cells was performed overnight at 56°C. After overnight hybridization, to minimize the amount of unhybridized probes, cells were washed three times with formamide followed by one wash with RNAse A, and then one wash with RNAse-free buffer. Depending upon the batch of reagents, the slides were coated with liquid emulsion (K5 Liquid Emulsion, Ilford Imaging, UK) and exposed for 10–15 days. After exposure, the slides were developed with Kodak D19 developer (Eastman Kodak Company, Rochester, NY) and fixed using the Ilford Hypam Fixer (Ilford Imaging, UK). The developed slides were then stained with Wright-Gimsa Stain (EM Diagnostics Systems, Gibbstown NJ) and mounted with permount. The probes, used for *in situ *hybridizations, were prepared by cloning a DNA sequence corresponding to the 5' untranslated region (5'-UTR), nucleotides 55–308, of HCV RNA into pGEM-T Easy vector (Promega Corp. Madison, WI). S^35^-labeled probes, complementary to the positive- or negative-strand of HCV-RNA, were generated by *in vitro *transcription in the presence of a ^35^S rUTP (Amersham Biosciences, England) using the appropriate RNA polymerases as supplied by the manufacturer (Promega Corp. Madison, WI) and purified through Sephadex G50 [11].

### Detection of HCV-RNA by fluorescence microscopy

An indirect immunofluorescence (IF) assay was used [11]. Cells were washed for 10 minutes three times with phosphate-buffered saline (PBS), resuspended in PBS, deposited on Teflon-coated slides, air-dried, and fixed in cold acetone for 10 minutes. Patients' heat-inactivated sera (56°C for 30 minutes and then clarified by centrifugation) was added to the fixed cells, and incubated at 37°C for 40 minutes. They were then washed with PBS, air-dried, and stained with FITC-conjugated anti-human IgG for 40 minutes. The cells were again washed, air-dried, counter-stained with Evans Blue for 5 minutes and mounted with IF mounting solution.

### Kinetics of HCV production *in vitro *to determine the optimum day for harvesting positive-strand CIMM-HCV RNA

On day zero, a CIMM-HCV cell culture was taken out of liquid nitrogen, resuspended, separated into seven flasks of approximately 10^6 ^cells each, and fresh media was added to each flask. The initial concentration of the virus in the media therefore starts at zero viral particles. For each of the next seven days, one flask was harvested and assayed for the positive- and negative-strands of HCV-RNA using nested RT-PCR.

### Genotyping of CIMM-HCV RNA

RNA from cell culture supernatants was amplified via nested RT-PCR using the positive-strand RT-PCR assay primer set as described before. Products of the RT-PCR were cloned into the PCR 4.1 cloning vector (Invitrogen Corp. Carlsbad, CA). Plasmid DNA was isolated from individual clones and sequenced on an ABI 377 automated DNA sequencer using a Dye Terminator Sequencing Kit (Applied Biosystems, Foster City, CA).

### Purification of Immunoglobulin (IgG) from HCV infected patient sera

Serum from patient #081 was applied to an Affi-Gel II Protein A column (Bio-Rad Laboratories, Hercules, CA), and the IgG fraction was eluted. Purified IgG were concentrated by Microcon 50 columns (Millipore Corp., Billerica, MA) and stored at -20°C.

### Extraction of viral proteins from cell culture supernatants

Total proteins were precipitated from 1 ml of cell culture supernatant or patient serum with the TRI REAGENT (Molecular Research Center, Inc. Cincinnati, OH). The ethanol washed protein pellet was solubilized into 200–500 μl of 1% SDS by incubating at 55°C for 10 minutes. Any remaining insoluble subcellular particles were removed by centrifugation at 14000 × g for 10 minutes at 4°C. Proteins were quantified using the Bradford Protein Assay (Sigma-Aldrich Corp. St. Louis, MO) and frozen (-20°C).

### Dot-blot and Western blot analyses

For the dot-blot assay, 2 μl of various protein samples (undiluted to 10^-3^) were diluted to 25 μl using TBS and were dot blotted onto a nitrocellulose membrane (0.22 μ, Micron Separations Inc. Westboro, MA). For the Western analysis, proteins were separated by SDS-PAGE under non-reducing conditions and transferred to nitrocellulose membranes (Bio-Rad Labs). The membranes were blocked with 2% non-fat milk in 20 mM TBS, 500 mM NaCl, 0.02% Tween 20 for 1 hour. The samples were then incubated with purified IgG (1:1000 dilution) for 2–4 hours at room temperature. Antibody binding was detected by incubation with alkaline phosphatase-conjugated goat anti-human antibodies followed by color development (Bio-Rad) [15].

### Accession numbers of HCV sequences used for genotyping

The 5' UTR sequence was obtained using the 10.1 and 10.2 primers (Table 1), and has the accession number DQ010313. The partial sequence of the NS5B region was obtained using the C-anti and the reverse complement of C1A primers, and has the accession number DQ010314.

## Results

**Figure 2:**
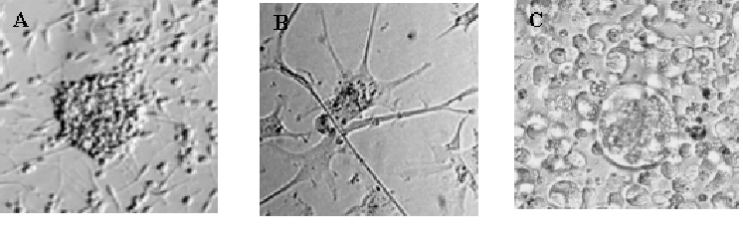
***In vitro *propagation of HCV in cultured cells**. Morphology of neuronal precursor cells infected with HCV. (**A**) T (telencephalon, suspension cells that grow in clumps and also can adhere to plastic), and (**B**) M (metencephalon, primarily adherent cells that develop neuronal processes). (**C**) Freshly transformed B-cells co-cultured with HCV infected macrophages. None of these cells have definitive cytopathic effects when compared with uninfected cells.

Nine years ago, we undertook to isolate infectious HCV from patients and to grow such isolates *in vitro*. Our initial experiments to develop an *in vitro *system of HCV replication were performed as previously reported by many investigators using a large variety of established cell lines comprising of various cell types [16]. These included human transformed liver cells in addition to Hela, CEM, H9, Jurkat, Molt 3, Molt 4, U937, P3HR1, Raji, Daudi, human foreskin fibroblast (ATCC, Bethesda, MD). All of these cell types could be infected by the reported methods, with the exception of human foreskin fibroblasts, which was uninfectable (Table 2). Results from these efforts did not prove to be reproducible for the sustained replication of HCV. Although we were able to detect positive and negative-strand (replicative) RNA for HCV in a few B-cells, liver cells, and monocytoid cells, none of these standard cell lines produced infectious HCV that could be transmitted into uninfected cells. These freshly infected cell cultures eventually became negative for HCV-RNA, while the uninfected cells grew. We now know from our experience that HCV behaves as a lytic virus, with up to 20% cell death in infected cultured cells. Infected B-cells form enlarged cells which eventually die without further replication (Fig. 2C). Cell line U937, despite its monocytic nature and the presence of detectable positive- and negative-strand HCV-RNA, had very low levels of viral RNA expression.

**Table 2:** Summary of HCV transmission experiments with various hematopoetic and liver cells

	**Short term**	**Long term**
**A. T-cells**^1^	+	-
**B. B-cells**^2^	+	+
**C. Monocytes/macrophages**^3^	+	-
**D. Neuronal precursors**^4^	+	+
**E. Liver cells**^5^		
Kupffer's cells	+	-
Hepatocyte	+	+/-

^1^T-cells isolated from human fetal chord blood.

^2^B-cells immortalized by infection with transforming EBV.

^3^Monocyte/Macrophages, adherent cells stimulated with PMA.

^4^Recently isolated neuronal cells from fetal human brain.

^5^Freshly isolated liver cells from liver biopsies. Kupffer's cells are liver macrophages and Hepatocytes are liver endothelial cells.

Because our initial experiments provided no significant improvement over the previously reported findings, we used a different approach for HCV isolation. We noted higher levels of HCV-RNA in infected macrophages compared to other infected cells. This was analogous to the infection of similar cells with human immunodeficiency virus (HIV-1) [17]. Therefore, we initiated the use of freshly isolated macrophages and other cells. We tested a variety of cell types from different origins for infectivity with HCV: endothelial cells from fresh fetal umbilical cord, mononuclear cells from fetal cord blood, CBMC, PBMC, and Kupffer's cells and hepatocytes from fresh liver biopsies. These freshly obtained cells were infectable and expressed both the positive- and the negative-strands of HCV-RNA.

Further experiments were designed that used macrophages as the intermediate host. The results from macrophage cultures were most encouraging. A number of researchers have previously used B-cells as a target [4,6]. Therefore, we decided to combine the macrophages with B-cells into one system. It also became apparent that in order to carry the transmitted virus for an extended period of time *in vitro*, long-lived B-cells were required. We opted in favor of freshly immortalized B-cells because they were free of various adventitious agents such as mycoplasma and other cellular contamination. Retransmissions were achieved by using the culture supernatants obtained from the macrophages and the B-cells prepared in our laboratories.

### Transmission of HCV isolates

In order to show that our system could be used to grow HCV for extended periods, we tested each isolate at regular intervals by RT-PCR and retransmission into fresh cells (Table 3). Due to the large number of samples that were tested, HCV isolation and long term replication were carried out in several phases: short term cultures (positive for HCV up to 10 weeks), medium term cultures (positive for 10–23 weeks), or extended term cultures (positive for over 23 weeks). Experiments using either human patient sera or PBMC were equally able to infect macrophages that could be used in cell-free transmission of HCV. We did not compare the levels of virus produced by these two methods. An example of a long term positive cell culture is isolate #081. This isolate was obtained from similarly numbered serum from donor #081. Isolate #081 has been maintained in culture for over one hundred thirty weeks. This is designated as the index isolate: CIMM-HCV. This isolate has been propagated in different cell types such as enriched B-cells, T-cells, and non-committed lymphoid cells obtained from fresh blood by both co-culture and cell-free methods. Serial transmissions to freshly transformed B-cells were performed by cell-free methods for further analysis (Figure 1B). The first transfer of HCV from macrophages to target cells is designated as T1. A transfer from the T1 culture to fresh target cells is designated T2. Transfers of isolates have been carried out as many as four times (T4), such as isolate PCLBT4. Cell culture supernatants were harvested at least every month and assayed for positive-strand HCV-RNA by nested RT-PCR analysis (Table 3). Nested PCR has been used as a diagnostic method by many researchers [18-20], and was used in order to eliminate false positives. Due to the consistently positive nested PCR and sequential biological transmission assays over a period of many months, the isolated HCV was considered to be replicating and infectious virus.

**Table 3:** History of HCV positivity for CIMM-HCV isolates^1^

	**Sample**				
**Month**	#081	112 A	112 B	112 AB	PCLB T4

1	-				
2	-				
3	+				
4	+	-	-		
5	+	+	+		
6	ND	+	+		
7	ND	+	+	-	
8	ND	+	+	+	-
9	ND	+	+	+	+
10	ND	ND	ND	ND	-
11	ND	-	-	ND	-
12	ND	+	+	ND	+
13	ND	+	+	ND	+
14	ND	+	+	ND	+
15	ND	+	+	-	+
16	ND	+	+	+	+
17	ND	+	+	+	+
18	ND	+	+	+	+
19	ND	+	+	+	+
20	ND	+	+	+	+
21	ND	+	+	+	+
22	ND	+	+	+	+
23	ND	+	+	+	+
24	ND	+	+	+	+
25	-	ND	ND	ND	ND
26	+	ND	ND	ND	ND
27	+	ND	ND	ND	ND
28	+	ND	ND	ND	ND
29	+	ND	ND	ND	ND
30	+	ND	ND	-	-
31	+	ND	ND	+	+

^1^Each sample represents a monthly harvest of cell culture supernatants that were tested for the presence of human HCV positive-strand RNA and the cell free transmission to fresh target cells. Each individual sample was stored in liquid nitrogen at various time points throughout our testing. CIMM-HCV has been carried for over 12 months as a primary culture and over 31 months as a transmitted virus into other cell types including T-Cells (112A), B-Cells (112B), non-committed lymphoid cells (112AB) and 4^th ^serial transmission into immortalized cord B-cells (PCLB T4). Primary cells are the first B-cells infected with HCV isolated from the macrophages.

Our results suggest that there is no significant difference between using patient sera or PBMC as a source of the infectious agent, but there were no attempts made to quantitate the levels of infectious virus in the primary samples (serum or cells). Since only one cell producing infective virus can be enough to achieve transmission, both methods can be used to successfully culture HCV.

### Host range of HCV isolates

CIMM-HCV is maintained in one cell type: freshly transformed B-cells. In order to establish the host range of this isolate, a large number of cell types were tested for HCV propagation as described before. In addition to B-cells and macrophages, neuronal precursors could also be infected. These neuronal cells are very similar to macrophages, and they became a significant producer of infectious HCV (Table 4). Neuronal T cells grow in large non-adherent and adherent clumps and the M cells are generally adherent and form neuronal cell-like processes. They survived HCV infection better than B-cells in terms of cell viability (Figs. 2A and 2B). Cell-free CIMM-HCV was transmitted to our two neuronal cell types, T (telencephalon) and M (metencephalon), which subsequently showed replication of transmissible infectious virus (experiment 244). Virus from these cells was subsequently transmitted to fresh T and M neuronal cell cultures in experiment 248 and from 248 to 260 (Table 4). These retransmissions were similar to the ones performed for B-cells (Table 3). Infections of neuronal cells were repeated several times with similar results with respect to HCV production. We have since transmitted this HCV from experiments 260 to 273 and 273 to 277 (data not shown).

**Table 4:** Transmission of human HCV in neuronal precursor cells^1^

	**Month**						
**Sample**	1	2	3	4	5	6	7

#081	+	+	+	+	+	+	+
244 M	-	+	+	+	+	+	+
244 T	-	+	+	+	+	+	+
248 M	-	+	+	+	+	+	+
248 T	-	+	+	+	+	+	+
260 M	+	+	+	+	+	+	+
260 T	+	+	+	+	+	+	+

^1^The neuronal precursor cells were isolated from human fetal brain by Dr. Olag Kopyov (see acknowledgment). They were designated T (telencephalon, suspension cells) and M (metencephalon, adherent cells). Each sample represents a monthly harvest of cell culture supernatant that were tested for the presence of positive-strand HCV RNA.

### Testing the HCV isolation system using additional patients

In order to take advantage of the system developed in our laboratories, we obtained 156 samples from patients who volunteered to donate their blood. Of these, 151 were peripheral blood specimens from HCV infected patients and 5 were from uninfected controls. All specimens were acquired with the approval of the Institutional Review Board (IRB) and donors' informed consent. The HCV-infected specimens were obtained from 109 Caucasians, 37 Hispanics and 5 African Americans. The uninfected controls were from 2 Caucasians, 2 Hispanics, and 1 African-American. The participants included 108 males and 48 females. All specimens were freshly processed within an hour of blood drawing. Repeat samples were obtained from 77 of the original patients in order to confirm our initial results. Thirty-three of these 151 HCV-infected patients were co-infected with HIV-1, and the remainder of the donors had hematological malignancies or other cancers. HCV was isolated with 75% efficiency from these 151 specimens. In the case of co-infected patients, the failure to isolate HCV was commonly due to rapid cell death. No HCV was ever isolated from the 5 uninfected controls. This high rate of isolation of HCV shows that this system is useful in obtaining HCV from a variety of individual patients for further analysis.

### Determination of optimum day for harvesting HCV for RNA extraction

**Figure 3:**
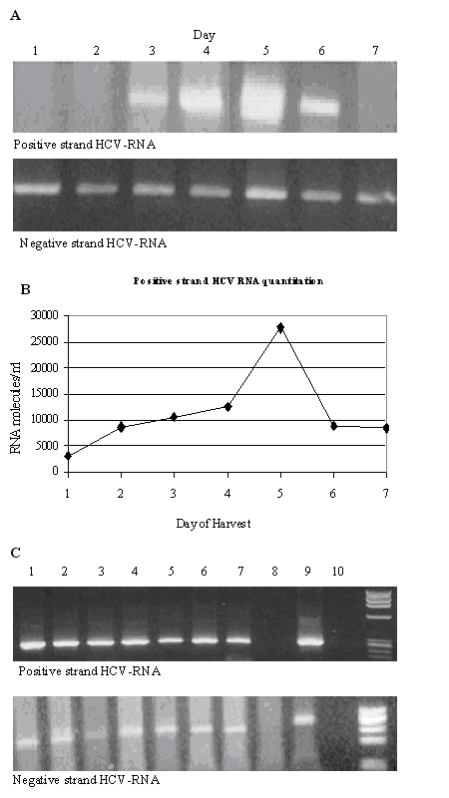
**Detection of positive- and negative-strand HCV-RNA in infected cell cultures via RT-PCR**. Posititve strands were assayed using the cell culture supernatant while the negative strands were assayed using total RNA purified from the cells. (**A**) Determining the optimum day to harvest HCV for RNA extraction and analysis. Approximately one million cells of culture #081 were divided into seven flasks and incubated. One flask was harvested on each of the following days and assayed for positive and negative strand RNA. (**B**) Quantitation of molecules of positive-strand HCV-RNA per ml of cell culture supernatant via real-time RT-PCR. (**C**) Positive- and negative-strand HCV-RNA in different cells infected with CIMM-HCV. Lane 1 CIMM-HCV, lane 2 T-cells (112 A), lane 3 B-cells (112 B), lane 4 non-committed lymphoid cells (112 AB), lane 5 the 4^th ^serial transmission into immortalized cord B-cells (PCLB T4), lane 6 T-cells (200 A), lane 7 B-cells (200 B), lane 8 uninfected B-cells, lane 9 HCV infected patient serum, and lane 10 negative PCR control.

In order to determine the optimum day for harvesting the highest accumulation of positive-strand RNA, the kinetics of HCV production was measured using nested PCR. For each of the next seven days, flasks were harvested and assayed for the positive- and negative-strands of HCV-RNA using nested PCR. An example of our results is shown in Fig. 3A. While day 5 showed the greatest accumulation of positive-strand of HCV-RNA, the levels of the negative-strand inside the cells on all seven days remained unchanged (Fig 3A). There was no significant increase in cell numbers during the experiment.

In an experiment performed simultaneously, the positive-strand HCV-RNA in the cell culture supernatants was analyzed quantitatively by real-time RT-PCR. As expected, on day zero there was no measurable HCV-RNA. On day one, the measurable number of copies of HCV-RNA was 3,200, which increased during the experiment to approximately 27,000 copies per ml on day 5 and then decreased from thereon (Fig. 3B). This data was consistent with the pattern obtained using the nested RT-PCR assay shown in Fig. 3A. Note that the data for Figures 3A and 3B are from using the same samples. The optimum day of harvesting this isolate of HCV was on day 5. Other isolates have produced similar growth curves (data not shown).

Seven isolates were tested by nested RT-PCR to show that the results from Figure 3A were reproducible. The presence of the expected PCR products demonstrated that on day 5, both positive- and negative-strands of HCV-RNA were present in our system (Figure 3C). This experiment shows both replication and extracellular production of the virus. This indicates that harvesting RNA on day 5 will permit reproducible results.

### Detection of HCV-RNA by *in situ *hybridization

**Figure 4:**
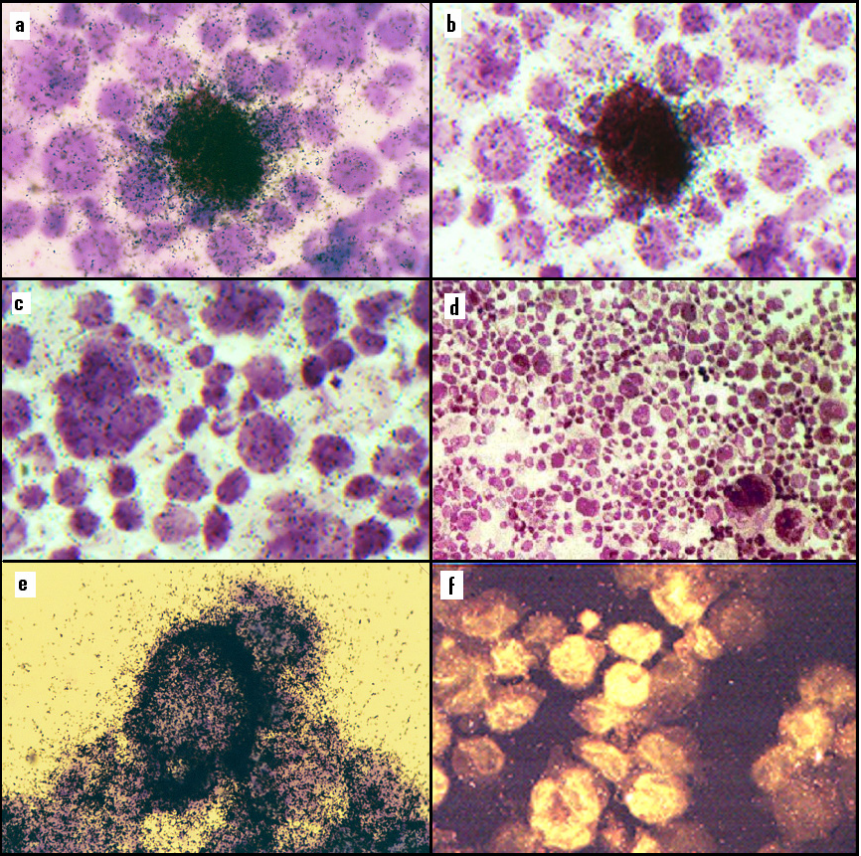
**Detection of HCV RNA in cultured cells by *in situ *hybridization with S^35^-labeled RNA probes and detection of HCV protein by fluorescence microscopy**. (**A, B**) Infected B-cells hybridized with labeled positive-strand RNA probe. (**C**) Freshly transformed uninfected B-cells showing no significant hybridization. (**D**) Picture of freshly transformed uninfected B-cells showing no significant hybridization and having a wider field of view than (C). (**E**) Infected B-cells hybridized with labeled negative-strand RNA probe. (**F**) HCV infected cells: PCLBT4 treated with human polyclonal IgG purified from the serum of patient 081 and stained with goat anti-human IgG conjugated with fluorescein isothiocyanate (FITC). PCLBT4 is the fourth consecutive transfer of HCV to freshly immortalized B-cells.

We analyzed our HCV infected cells by performing *in situ *hybridizations to visualize the percentage of infected cells and the locations of the HCV-specific strands [21]. The uninfected cells used as a control did not hybridize to either negative or positive strand probes (Figs. 4C and 4D). In all cases, the numbers of background grains were light. Hybridization with the probe for the positive-strand produced a halo-like appearance around the periphery of the infected cells (Fig. 4E). A strong signal for the negative strands of HCV-RNA was seen confined within the cells, possibly in the cytoplasm (Figs. 4A and 4B). Fluorescence microscopy of infected cell cultures showed a similar result (Fig. 4F). Although approximately 5% of the cells appeared strongly positive, this may have been an underestimate due to: (1) cell lysis of infected cells in culture; and (2) the loss of cells that attach to the filter cards used in preparing the cytospin slides. Hybridization to both the positive- and negative-strands of HCV-RNA suggests replication and production of HCV. Since most of the cells do not appear positive, the positivitity that was observed is not just a result of non-specific staining of cells. Results of the *in situ *hybridizations are consistent with the nested RT-PCR assay described above. A majority of the infected cells appear to be large; however, there were a significant number of smaller cells that also gave positive signals above background. By comparison, neither the enlarged cells nor the small ones in the control population showed any positive signal (Fig. 4C and 4D). We believe that the small, infected cells produce virus and probably progressively enlarge and die, as trypan blue dye exclusion tests showed that these cells eventually died. Similar phenomena are observed in human immunodeficiency virus (HIV) and HHV-6 infected cell cultures [22].

### Genotyping of the CIMM-HCV isolate

Based on sequence analysis, HCV has been classified into six major genotypes and a series of subtypes [23]. The highly conserved 5' untranslated region (5'-UTR), routinely used for RT-PCR detection of HCV-RNA, exhibits considerable genetic heterogeneity [24] and shows polymorphism between types and subtypes. This genetic heterogeneity of the 5'-UTR has been utilized for the genotyping of HCV [19,25-29], therefore, the 5'-UTR of CIMM-HCV was cloned and sequenced. Based on sequence homology searches, CIMM-HCV was similar to genotype 1a.

In order to spot check the genome of CIMM-HCV, we tested most of the previously published primers [30-33]. We, however, found that many of these primers did not lead to RT-PCR products from our isolate, including CD 2.10 [31], CD 5.10 [31], CD 5.20 [31], A5310 [33], and A6306 [33]. This may be due to the heterogeneity of HCV RNA [18]. It is also possible that parts of our isolate may differ significantly from the previously reported sequences. We have included here the sequence from part of the NS5B gene of CIMM-HCV, which is located near the 3' end of the genome. This sequence is most similar to HCV of genotype 1a/2a.

Although the culture system described here is capable of isolating HCV from approximately 75% of infected patients, this process may select more competent and infectious virus. Our analysis of sequences from the 5' UTR region shows in one case that the blood of a patient and the isolate in culture are both of type 1b. There were no significant differences in the sequences of the patient and the isolate in this region (Revie, Alberti, and Salahuddin, manuscript in preparation).

### Reactivity of the polyclonal IgG purified from infected patient sera

**Figure 5:**
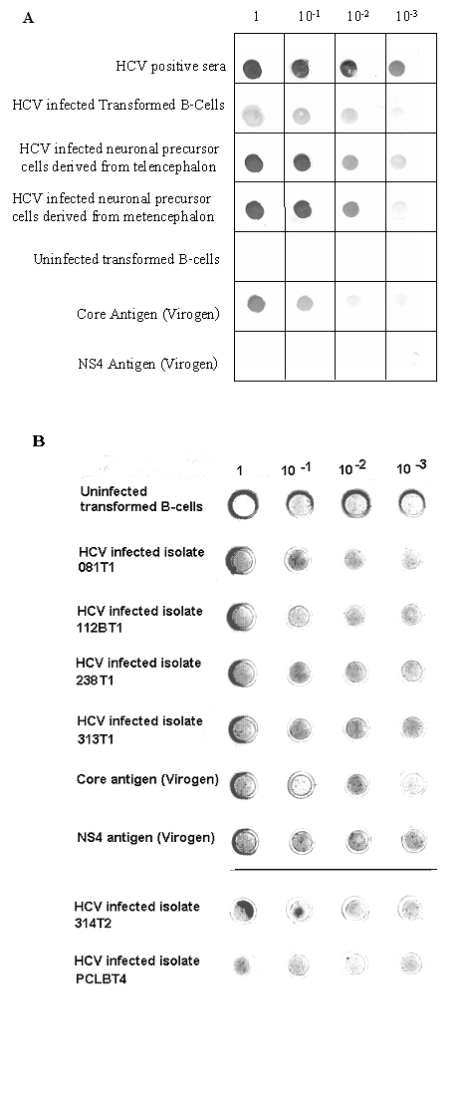
**Dot blot analysis of HCV proteins binding to IgG from patient sera**. HCV proteins were obtained from tissue culture fluid. Protein preparations were serially diluted (1, 10^-1^, 10^-2^, 10^-3^) and were dot blotted onto a nitrocellulose membrane. These blots were then treated with patient antibodies. The figure shows **(A) **Reactions of patient IgG against dilutions of IgG depleted patient sera, CIMM-HIV cell culture supernatants from different cell lines (HCV infected B-cells, HCV infected human neuronal precursor T and M cells), or commercial antigens (NS4 and Core antigen), or uninfected B-cells. **(B) **Reactions of patient IgG against dilutions of various HCV isolates grown *in vitro *as described before. All HCV isolates were from the first transfer to fresh B cells (T1) except for isolates 314T2 (second transfer) and PCLBT4 (fourth transfer). These infected cells have been in culture for varying periods of time, including over three years for PCLBT4.

To determine the reactivity of the purified polyclonal IgG, various dilutions of the total protein preparations from cell culture supernatants were analyzed. A positive reaction was noted with homologous serum proteins using CIMM-HCV obtained from the B-cell supernatant, supernatants from neuronal cells (from transmission experiment 260), and commercially available HCV core antigen (ViroGen Corp. Watertown, MA) (Fig. 5A). There was no reaction with NS4 as well as the uninfected cell culture supernatants. These results show that IgG purified from patient's sera specifically detects HCV virion proteins, particularly Core antigen, and that the virus grown in culture reacts with antibodies from patient's sera.

Six different independent HCV isolates (081T1, 112T1, 238T1, 313T1, 314T2, and PCLBT4) were tested against polyclonal antibodies from patient 238 using a dot blot (Figure 5B). This was performed in order to determine if these isolates reacted similarly to the previous experiment shown in Figure 5A. The patient antibodies reacted with all of these isolates, as well as to commercial Core antigen and NS4. The amount of undiluted NS4 used here was 2 μg. This shows that all of these HCV isolates are producing HCV proteins, and that even a fourth transfer (T4) of one isolate into freshly transformed B-cells still produces reactive HCV proteins (PCLBT4). Each of these isolates has been passaged in culture many times.

### Analysis of HCV proteins

**Figure 6:**
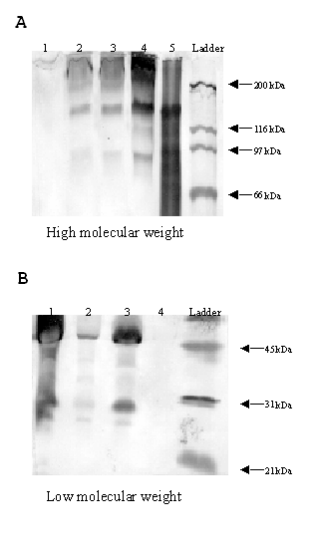
**Western blot analysis of HCV proteins under non-reducing conditions**. Proteins were isolated from cell culture supernatants. **(A) **Analysis of large molecular weight proteins from the cell culture supernatants of CIMM-HCV in various cell lines. Lane 1 uninfected transformed B-cells, lane 2 transformed B-cells infected with HCV, lane 3 neuronal precursor cells derived from the metencephalon infected with HCV, lane 4 total protein from HCV positive sera and lane 5 commercially engineered HCV core antigen (ViroGen). Other smaller bands in the Core antigen may be due to breakdown products or other contaminating proteins. **(B) **Analysis of low molecular weight proteins of CIMM-HCV cultured in various cell lines. Lane 1 total protein from HCV positive sera, lane 2 transformed B-cells infected with HCV, lane 3 neuronal precursor cells derived from the metencephalon infected with HCV, and lane 4 uninfected transformed B-cells.

The HCV genome encodes a polyprotein which is subsequently processed into a number of mature structural and nonstructural moieties [34]. In order to determine whether the replicating CIMM-HCV was producing major HCV proteins, Western blot analyses using non-reducing conditions were performed. The polyclonal IgG detected a series of proteins (i) in the HCV positive patient sera and (ii) in the infected cell culture supernatant (Figs. 6A and 6B). Proteins of 140, 75, 50, 37, 32, 27 and 25 kDa were detected in these samples. The polyclonal IgG also gave a positive reaction with the commercially obtained recombinant core antigen (lane 5, Fig 6A). This core antigen has β-galactosidase fused at the N-terminus and is thus approximately 140 kDa in size, as reported by the manufacturer.

There are two highly glycosylated envelope proteins, E1 (32 and 35 kDa) and E2 (70 kDa) [35-39]. A band at approximately ~ 140 kDa was seen in all of the HCV isolates (Fig. 6A). This band has been seen by other researchers [40,41], and may have resulted from the multimerization of core, E1 and E2, or homodimerization of E2. The E1 and E2 proteins are known to form non-covalently linked heterodimers under non-reducing conditions [36,40,42]. The Core and E1 proteins also bind to each other [43,44], and. possibly form HCV protein and host cellular protein complexes as well.

Proteins in the 32 and 37 kDa range were also detected in the Western blots (Fig. 6B). These bands are consistent with the known sizes of E1. An approximately 75 kDa protein was also detected in all of the infected samples that were analyzed (Fig. 6A). This protein corresponds to the known molecular weight of E2.

In all the HCV isolates that were tested, a major protein band of approximately 50 kDa was seen (Fig. 6B). This was perhaps due to the incomplete processing of the precursor polyprotein [45]. Since the band was present only when protein purified from infected cell culture supernatants were used for the analyses, the band is therefore related to HCV proteins.

Bands of approximately 25 and 27 kDa were also detected on the Western blots (Fig. 6B). The host cell signal peptidases cleave the N-terminal region of the precursor polypeptide to produce the HCV core protein [37,46,47]. The HCV core protein is reported to range between 16 and 25 kDa in size. However, it is possible that the size differences that have been previously reported may be due to differences in processing of the HCV core protein [37,45,48,49]. We believe that the Core antigen, as expressed by our isolates of HCV, may be larger than has been previously described.

CIMM-HCV grown in three different cell lines produced the same pattern of bands. Although we were using polyclonal IgG extracted from patient sera, we detected HCV proteins that have been reported by other researchers.

## Discussion

Our cell culture system comprises freshly prepared macrophages and immortalized B-cells for the isolation of HCV. The isolations are performed using both co-cultures and cell-free methods. Freshly prepared macrophages in our system are essential as an intermediate host for HCV isolation. We have not explored the mechanism of infection or replication of HCV, but macrophages from a variety of sources appear to have a positive role in this process. It is possible that macrophages either concentrate the HCV particles or modify the HCV sufficiently to enable them to infect other cell types. For example, changes in the glycosylated envelope proteins E1 and E2 [36,38,39] could affect the infectious capability of the progeny virus, as well as define its host range. In bacteria, infecting phage can have their DNA modified by the host modification system. This allows the phage to escape the restriction system, thus enabling better infection of new hosts. It is therefore not too difficult to imagine that various types of animal cells could produce modified versions of infecting viruses. These viruses may preferentially infect different cell types. It is also possible that the macrophages reduce or eliminate defective HCV that are found in the patient's blood that may interfere with starting a productive culture system. The macrophages would thus be acting as a gatekeeper, only allowing intact HCV to be cultured and disallowing defective ones that could interfere with the culturing.

As stated before, we discovered that neuronal precursor cells can be infected with CIMM-HCV and, in turn, are significant producers of infectious virus. We have repeatedly transmitted several HCV isolates into these neuronal cells. These cells are similar to other macrophages both in staining characteristics and in functional assays. They are growth factor dependent and grow well *in vitro*, and have been in culture for over two years. Macrophages from other sources, e.g. Kupffer's cells from liver, get infected, but after a few weeks gradually lose virus production. HCV-RNA, however, can be detected for several weeks. This loss of virus production may be related to the maturation, cytostasis, and eventual death of Kupffer's cells. Similar experiments were performed with freshly cultured endothelial cells obtained from human umbilical cord, because these are related to hepatocytes from liver, which are also endothelial cells. The results from these cells also showed a pattern of virus production similar to Kupffer's cells.

For almost all human viruses, there is a well-observed phenomenon of asynchrony of expression, with a few notable exceptions such as HIV. For human Herpes viruses, no matter which host cell they use, only a minority of cells produce replicating viruses [50,51]. We do not know why less than 5% of the cells in our system are productive. Human immunodeficiency viruses such as HIV-1 show active replication in less than 20% of infected T-cells if freshly isolated leukocytes are used [52]. The system we have described uses all freshly isolated cells. It is very likely that a specific receptor may be the limiting factor in determining the number of infectable cells.

We determined that the amount of HCV-RNA in the cell culture supernatant rises until day 5, then gradually declines. Therefore, the fifth day after subculturing is optimum for harvesting of HCV-RNA. As shown in Figure 3C, harvesting RNA on day 5 allows reproducible detection of HCV-RNA. Changes in the overall levels of HCV-RNA may reflect the sum of the RNA production and RNA destruction in culture. This observed periodicity in the positive-strand therefore, may be due to: (1) slowing of the replication process of the infected cells or from production of an inhibitor *in situ*; or (2) lysis of infected cells causing destruction of the virus and its RNA, e.g. by released proteases and ribonucleases, or (3) both of the above mentioned possibilities. The measured level of the negative strand in the cells remained stable. Since the number of cells and the percentage of cells that are infected don't change during the culturing of the virus, the observed stability of the negative-strand inside the cells is understandable.

Although the majority of patient samples described above are of type 1b, our analysis of CIMM-HCV sequences show it is type 1a. In the 5'UTR region there is very little sequence difference between types 1a and 1b. Changing of one base can cause the sequence to more closely resemble type 1b. It is possible that only a small subset of HCV in a patient is actually infectious, and therefore our system best typed it as type 1a. A complete sequence of the CIMM-HCV genome will reveal its identity. It is possible that a chronic infection in patients may develop mutants that resemble another type. However, we have additional isolates that are consistent with other genotypes such as 1b (data not shown). The sequences that we have reported here indicate that both ends of the viral genome are present in our cultures. We are currently in the process of sequencing the entire CIMM-HCV genome in order to better determine its genotype as well as to compare it to the currently published HCV genomes.

Since this is the first *in vitro *system for culturing HCV, we have been able to make initial observations regarding replication of the virus. Further studies related to HCV replication and pathogenesis are in progress. The *in situ *hybridization results seen in Figure 4 suggest that the positive strand of HCV is synthesized at or migrates to the plasma membrane and that the negative strand remains in the cytoplasm. This observation can only be made in a dynamic system with actively replicating virus. This suggestion is supported by recent reports that RNA-dependent RNA polymerase contains a transmembrane segment which is anchored in the membrane [53]. Non-structural proteins and positive strand RNA have also been found associated with the plasma membranes [54]. These results suggest that the site where HCV is fully assembled is probably in or near the plasma membrane of the infected cells. Probably HCV-RNA is synthesized in the cytoplasm and migrates to the plasma membrane for the final assembly. The completed virion is then released into the extracellular space.

We believe that a chronically infected patient makes antibodies to their own virus. Hence, the polyclonal IgG is specific for that virus. This is confirmed by our dot blot analysis of several different HCV isolates. Since these isolates had undergone as many as four separate transfers into fresh cells and many serial passages, the system that we describe here maintains stable isolates in culture, producing HCV-specific protein.

Similarly, data from Western blot analysis of the HCV proteins in the cell culture supernatants show that all of the expected major structural proteins are present. No specific antibody bindings were seen in the samples from uninfected cell culture supernatants. Taken together, these results suggest that there is production of HCV specific proteins in CIMM-HCV infected cell cultures. This data shows that the virus that was grown in culture contains epitopes of all of the major structural proteins that react with antibodies purified from the patient. This is strong evidence that the virus grown in culture is not significantly different from the HCV growing in the patient. In addition, one isolate that was cultured in several different cells lines showed a consistent pattern of bands on Western blots. This is additional and strong evidence that the bands result from HCV proteins. We have concluded that the replicating HCV is a stable virus.

In addition to the molecular analysis which establishes that our cells are producing HCV virions, the serial transmission of HCV to fresh uninfected cells via cell-free culture supernatants establishes biological evidence of infectious virus (Figure 1B: PCLBT1-PCLBT4). Since this virus is infectious *in vitro *and all of the major proteins appear to be present, the virus that has been grown in culture most likely contains the entire genome. We do not believe that there is a large amount of defective HCV present in our system. Although our standard assay is to amplify the 5' UTR region, we have been able to obtain sequences from other regions of the genome. This, coupled with the evidence for the presence of the major HCV structural proteins, is strong evidence that the entire genome is present. In addition, we have been able to use the HutLA2 primer (Table 1), which is complementary to a region near the 3' end of the positive strand, to produce cDNA. Using our standard primers from the 5' UTR region to amplify the cDNA, we were able to detect HCV positive-strand RNA (data not shown). This suggests that a significant proportion of the RNA present contains both the 3' and 5' ends of the genome. In addition, work in progress suggests that a representative virus population grows in this system, suggesting that a particular genotype is not selected (Revie, Alberti, and Salahuddin, manuscript in preparation).

Our system has allowed us to reproducibly isolate HCV from a majority of patients and in a few cases these cell cultures have been carried for over 130 weeks. Our ability to detect HCV for these extended periods shows that we are not just detecting virus that has been diluted from the initial sample. For the initial infection, 50 μl of serum was added to 2 ml of media, and then the media was changed once a week for months. A conservative estimate of the dilution of the virus at 130 weeks would be over 10^130^-fold. The amount of HCV produced from this system was sufficient to conduct biological, molecular, and immunological investigations. We are continuing to pursue other investigations in this field.

The analogy between macrophage-initiated *in vitro *propagation of HIV and HCV is rather remarkable. The dendritic cell-specific ICAM-grabbing non-integrins (DC-SIGN) can bind HIV, and protect it for protracted periods to concentrate and deliver the virions to cause infection of T-cells in *trans *with high efficiency [55,56]. The structural basis for selective recognition of oligosaccharides on virion envelope proteins by DC-SIGN and DC-SIGR may indeed be a common pattern by which HIV and HCV are concentrated for *in vitro *transmission to their respective susceptible cells [57,58]. Unlike CD4 for HIV, the HCV receptor CD81 is currently a subject of serious discussions [58-61].

We would like to point out that our system allows us to produce significant quantities of HCV, which made these studies possible. Having a reliable and long-term growth system for HCV in cell culture will facilitate *in vitro *studies and also aid in the production of rational drugs and vaccines. This culture system will, therefore, allow researchers in the field of HCV and liver disease to perform a wide variety of further analyses that can help in understanding the life cycle of HCV and the mechanisms of pathologies induced in human hosts.

## Declaration of Competing Interests

All intellectual rights are reserved by the California Institute of Molecular Medicine (CIMM), and all aspects of this work were performed by CIMM. There are no competing interests between California Lutheran University or any other body and CIMM.

## Authors' contributions

SZS and RK performed the biological work and the isolations, transmissions, and retransmissions of HCV. JGP, ASK, CG and RR performed the clinical work, recruitment of patients, and procurement of specimens. DR, RSB, DB, and NC performed the molecular work.
